# Supplementary material for: Complex‐centric proteome profiling by SEC‐SWATH‐MS
Source: Mol Syst Biol. 2019 Jan 14;15(1):e8438. doi: 10.15252/msb.20188438 (PMC6346213; doi:10.15252/msb.20188438)
Supplement: Supplementary file 8 — Dataset EV7 [file MSB-15-e8438-s008.zip › feature_plots_string/Q9P086.pdf]

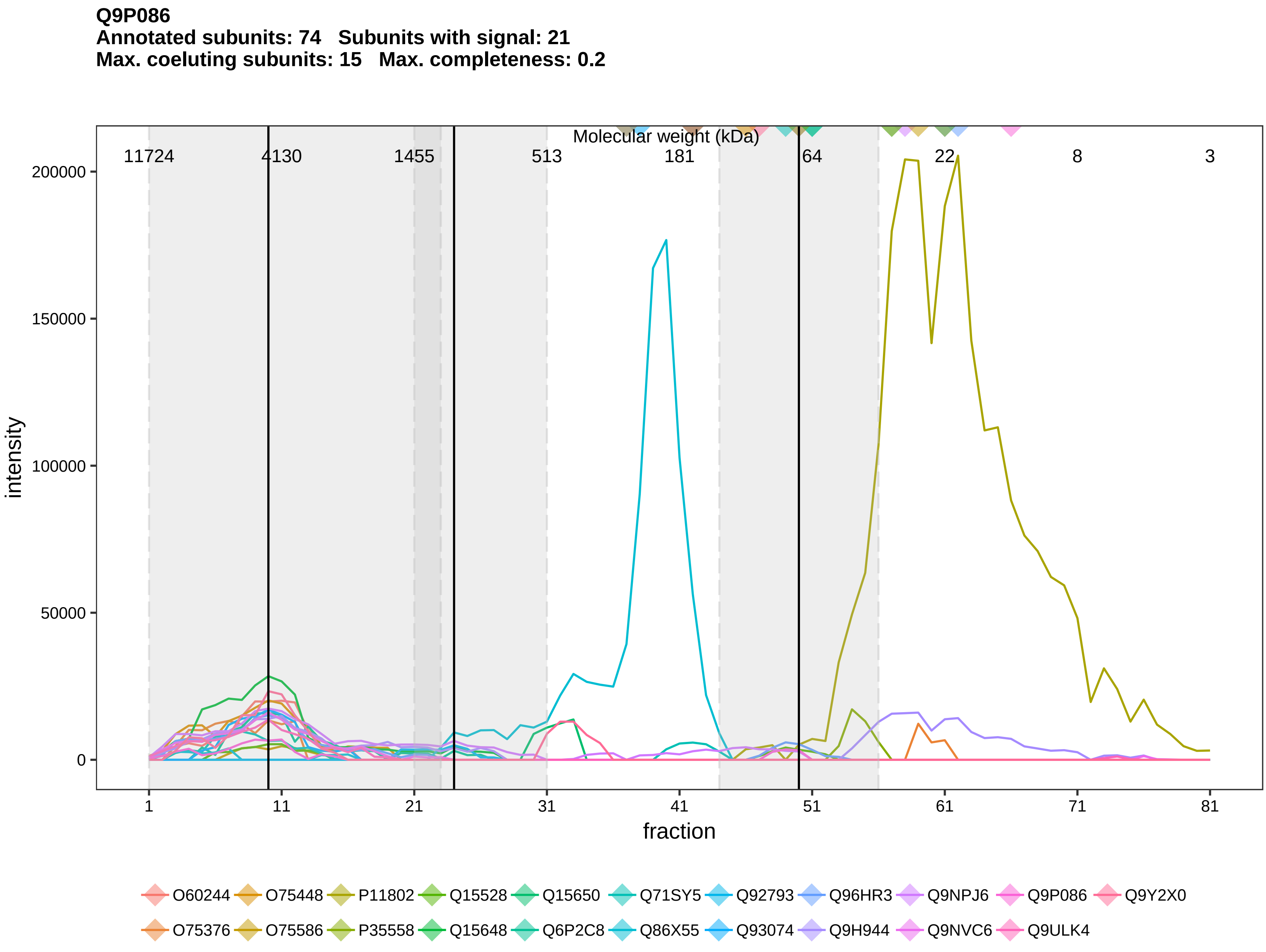
Q9P086
Annotated subunits: 74 Subunits with signal: 21
Max. coeluting subunits: 15 Max. completeness: 0.2
Molecular weight (kDa)
11724
4130
1455
513
181
64
22
8
3
intensity
200000
150000
100000
50000
0
fraction
1
11
21
31
41
51
61
71
81
O60244
O75448
P11802
Q15528
Q15650
Q71SY5
Q92793
Q96HR3
Q9NPJ6
Q9P086
Q9Y2X0
O75376
O75586
P35558
Q15648
Q6P2C8
Q86X55
Q93074
Q9H944
Q9NVC6
Q9ULK4
